# Magnetic ground state of SrRuO_3_ thin film and applicability of standard first-principles approximations to metallic magnetism

**DOI:** 10.1038/s41598-017-04044-6

**Published:** 2017-07-05

**Authors:** Siheon Ryee, Myung Joon Han

**Affiliations:** 1 Department of Physics, Korea Advanced Institute of Science and Technology (KAIST), Daejeon, 305-701 Korea; 2 KAIST Institute for the NanoCentury, KAIST, Daejeon, 305-701 Korea

## Abstract

A systematic first-principles study has been performed to understand the magnetism of thin film SrRuO_3_ which lots of research efforts have been devoted to but no clear consensus has been reached about its ground state properties. The relative *t*_2*g*_ level difference, lattice distortion as well as the layer thickness play together in determining the spin order. In particular, it is important to understand the difference between two standard approximations, namely LDA and GGA, in describing this metallic magnetism. Landau free energy analysis and the magnetization-energy-ratio plot clearly show the different tendency of favoring the magnetic moment formation, and it is magnified when applied to the thin film limit where the experimental information is severely limited. As a result, LDA gives a qualitatively different prediction from GGA in the experimentally relevant region of strain whereas both approximations give reasonable results for the bulk phase. We discuss the origin of this difference and the applicability of standard methods to the correlated oxide and the metallic magnetic systems.

## Introduction

The study of transition-metal oxide heterostructures and thin films has been an exciting field of research^1–5^. As the controlled synthesis and the combination of multiple ‘mother compounds’ become feasible, new possibilities can be realized to create the unique functionality and another angle to look at a long-standing problem from a different perspective. Simultaneously, however, it requires the further technological developments; not only for synthesizing but also for characterizing the materials. For example, the site dislocation, interface sharpness, stoichiometry and the oxygen vacancy have often been issues to understand the observed phenomena and the discrepancies between different measurements^3–5^.

Partly in this regard, first-principles approach has been playing a central role in this field from the early stage^3, 4^. Due to its capability of independent simulation, first-principles calculation not just provides the better understanding of experiments, but is also useful to predict or suggest a new system to have desired properties^4, 6–10^. As for the computation methods, however, there are several limitations and challenges to be overcome especially when the ‘(on-site) electronic correlation’ becomes important^11, 12^.

In this paper we study the magnetism of SrRuO_3_ (SRO113) thin film. First of all, it is an interesting system from both fundamental science and application point of view^13–24^. Its possible applications, for example, to the bottom electrode and field-effect devices have been discussed^13^. Further, a fundamental understanding of its magnetic properties is still controversial^15–24^. While the ferromagnetism of SRO113 has traditionally been discussed from Stoner’s picture^13, 16, 25^, recent studies provide new insights^19, 26, 27^. The relationship to its cousins (*e*.*g*., CaRuO_3_, Sr_2_RuO_4_, Ca_2_RuO_4_, *etc*), which show fairly different characteristics including antiferromagnetic insulating (AF-I) phase and superconductivity^28, 29^, should also be clarified. Thin film SRO113, in particular, exhibits the intriguing thickness-dependent magnetic and metal-insulator transition^15–18^. The nature of this transition is far from clear. For example, there is an obvious discrepancy in the previous reports regarding the critical thickness, and the ground state magnetism has not been clearly identified^15–18, 20–24^.

Here we perform a systematic and comparative investigation of SRO113 based on the standard first-principles computation methods. We elucidate the controversies in the previous calculations^16, 20–24^ and partly resolve the unsettled issues including the thickness and strain dependent transition of magnetic and electronic properties^15–18, 20–24^. The role of strain, correlation and lattice distortions are clarified. We further analyze the applicability of primary approximations, namely, LDA (local density approximation) and GGA (generalized gradient approximation), to metallic magnetism. Landau free energy analysis and the magnetization-energy-ratio plot clearly show the different tendency of favoring the magnetic moment formation. In case of thin film, as a result, LDA gives a qualitatively different prediction from GGA in the experimentally relevant region of strain whereas both approximations give reasonable results for the bulk phase. We discuss the origin of this difference and the applicability of standard methods to the correlated oxide and the metallic magnetic systems. Our work carries important information for future study especially when the experimental information is severely limited just as in the thin film or heterostructure.

## Results

### Bulk

SRO113 is a ferromagnetic metal (FM-M) with Curie temperature of 160 K and magnetic moment (*M*) of 1.1–1.7 *μ*_B_/f.u.^13^. Orthorhombic crystal structure is stabilized below 850 K with GdFeO_3_-type distortion^13^. To investigate SRO113, we first optimized the lattice parameters and the result is in good agreement with the previous studies (Table 1)^20, 21, 30, 31^. A well-known trend of the underestimated/overestimated lattice parameters by LDA/GGA is clearly seen. The deviation from experimental lattice values is within ±1.2%. Rotation and tilting angles calculated by LDA and GGA are slightly overestimated (within ~1.6–2.2°) in comparison with the experimental value of 8.61° and 8.60°, respectively.

**Table 1:** The calculated lattice constants, Stoner *I*, *I*N(E_F_) and magnetic moments for c-SRO113 and o-SRO113 by LDA and GGA.

System	Type	*a* [Å]	*b* [Å]	*c* [Å]	Stoner *I* [eV]	*I*N(E_F_)	*M* [*μ*_B_/f.u.]
c-SRO113	Exp.	3.92	3.92	3.92	—	—	—
	LDA	3.890	3.890	3.890	0.44	1.28	1.04 (0.67)
	GGA	3.988	3.988	3.988	0.51	1.86	2.00 (1.38)
o-SRO113	Exp.^67^	5.5670	5.5304	7.8446	—	—	—
	LDA	5.4998	5.4852	7.7503	0.46	1.27	0.82 (0.56)
	GGA	5.6313	5.6221	7.9500	0.53	1.75	1.99 (1.40)

For comparison, the experimental values are also presented. The experimental lattice constants of c-SRO113 are taken to be the pseudocubic values of o-SRO113. The *M* values in parentheses represent the magnetic moments at Ru sites.

Ferromagnetic ground state is well reproduced by both LDA and GGA with the optimized and experimental lattice parameters. The calculated moments are summarized in Table 1 being consistent with the previous studies^20, 30, 31^. About 70% of total moment resides at Ru-site and the other amount comes from O-site due to the hybridization^20, 25^. GGA moment is larger than LDA by about factor of two. Here we note this unusually large difference of the calculated moments by two different exchange-correlation (XC) functionals. It may indicate either that GGA overestimates the ferromagnetism or that LDA underestimates it. This difference becomes critical when one tries to predict the ferromagnetic instability in the thin films for which the experimental detections are largely limited. This point will be highlighted and further discussed later in this paper.

The GGA trend of stronger (than LDA) preference for ferromagnetic solutions is also found in the magnetic stabilization energy: Δ*E*_LDA_ = Δ*E* ^FM^ − Δ*E* ^PM^ = −7.8 meV/f.u. and Δ*E*_GGA_ = −106 meV/f.u. for o-SRO113 (orthorhombic SRO113). For c-SRO113 (cubic SRO113), Δ*E*_LDA_ = −14 meV/f.u. and Δ*E*_GGA_ = −102 meV/f.u.

**Figure 1:**
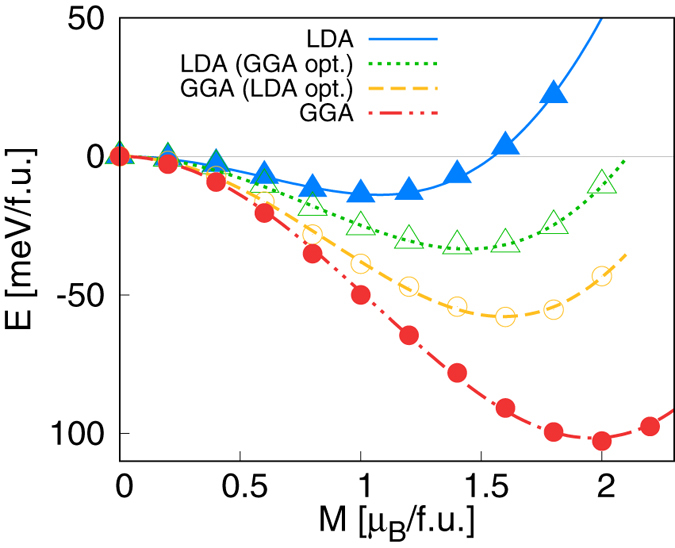
FSM calculation result of total energy for c-SRO113 as a function of *M*. The lines are obtained from the fitting to the Landau free energy. The open triangles (green) and open circles (yellow) refer to the LDA results with the GGA-optimized structure and the GGA results with the LDA-optimized structure, respectively. The nonmagnetic state is set to be the zero reference energy.

To have further understanding, we performed the fixed spin moment (FSM) calculations which have not been reported before in these materials. Our result is summarized in Fig. 1. The aforementioned magnetic behavior, namely the enhanced (suppressed) ferromagnetism by GGA (LDA), is again clearly manifested in this plot (see blue and red lines in Fig. 1). Note that this is not just the effect from the different lattice constants given by GGA and LDA optimization. Even when the same crystal structures were used, GGA total energy more favors the ferromagnetic solution than LDA; compare the blue-solid line (filled triangles) with the yellow-dashed line (open circles), and compare the green-dotted line (open triangles) with the red-dashed-dotted lines (filled circles). It is also noted that the GGA-optimized crystal structure is more favorable to ferromagnetism than the LDA structure; compare the blue solid line (filled triangles) with the green dotted lines (open triangles), and compare the yellow-dashed line (open circles) with the red-dashed-dotted lines (filled circles).

The ferromagnetism in ruthenates has been understood traditionally based on the Stoner model^13, 16, 25^. Therefore, it would be fruitful to estimate the Stoner parameter (*I*) from our FSM calculation results. Landau free energy can be written as,

1 $${\rm{E}}(M)=E\mathrm{(0)}+({a}_{2}/2){M}^{2}+({a}_{4}/4){M}^{4}+\cdots ,$$

where E(*M*) is the total energy as a function of magnetic moment (Fig. 1). The uniform spin susceptibility is given by

2 $${\chi }^{-1}={\frac{{\partial }^{2}{\rm{E}}}{\partial {M}^{2}}|}_{M=0}={a}_{2}=\frac{1}{2}(\frac{1}{{\rm{N}}({{\rm{E}}}_{{\rm{F}}})}-I),$$

where N(E_F_) is the non-spin-polarized density of states (DOS) per spin at Fermi level (E_F_). The calculated Stoner *I* and *I*N(E_F_) are presented in Table 1. The LDA result is in good agreement with the previous study^25^. Both LDA and GGA results satisfy the Stoner’s criterion of ferromagnetism and the calculated magnetic moments are in reasonable range consistent with experiments. The calculated *I* and *I* N(E_F_) values by GGA are larger than those of LDA by ~17% and ~40%, respectively. Namely, the stronger preference for FM solution by GGA (than LDA) can also be seen in these parameters. We once again emphasize that this cannot be attributed to the lattice effect. It is rather directly related to the parameterization of XC functional itself and thus requires further understanding at the more fundamental level. Our result demonstrates that the special care needs to be paid in ruthenates and related systems when one uses first-principles methods even within the standard XC approximations.

### Undistorted thin films: 1*a* × 1*a* lateral unitcell

Now let us turn our attention to the thin film. The previous calculations and experimental results are not quite consistent with one another regarding, for example, the thickness-dependent magnetic states on SrTiO_3_ substrate^13, 15–18, 20–24^. Since SRO113 thin film and its magnetic property have been studied often within 1*a* × 1*a* lateral unitcell (uc)^16, 20^, we focus on 1*a* × 1*a* films before considering the effect of tilting/rotational distortions of RuO_6_ octahedra.

**Figure 2:**
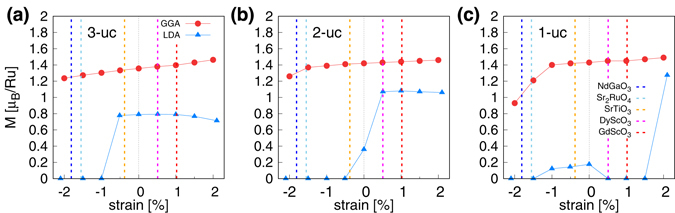
The calculated Ru moments of (**a**) 3-uc, (**b**) 2-uc, and (**c**) 1-uc thick c-SRO113 by LDA (blue solid lines; triangles) and GGA (red solid lines; circles) as a function of strain. The zero strain is set to the optimized lattice parameters of the bulk structure by each XC functional (Table 1). The vertical dashed lines represent the strains realizable by NdGaO_3_ (blue), Sr_2_RuO_4_ (light blue), SrTiO_3_ (orange), DyScO_3_ (magenta), and GdScO_3_ (red) as substrates.

Our results are summarized in Fig. 2. The calculated magnetic moments by GGA (red circles) and LDA (blue triangles) are presented as a function of strain. Figure 2(a–c) shows the result of the 3-uc, 2-uc, and 1-uc thick SRO113 films, respectively. In the 1*a* × 1*a* lateral uc, antiferromagnetic spin order can not be simulated and the finite moment indicates the ferromagnetic order as in the previous studies^16, 20^. It is noted that GGA more prefers the ferromagnetic solution than LDA, which is the same tendency as observed in the calculations of bulk SRO113.

One general feature found in Fig. 2 is that the ferromagnetism is suppressed by compressive strain. Both LDA and GGA predict the zero moment or paramagnetic phase under the large enough compressive strain. Importantly, however, the difference between GGA and LDA leads to a different prediction for the existence of ferromagnetic order and the critical value of strain below (above) which the magnetic moment or ferromagnetic order vanishes (sets in). In GGA, the ferromagnetic order is robust for 3-uc, 2-uc, and 1-uc films, which is significantly different from the LDA predictions of ≤−0.5 (3-uc)–0.0% (2-uc).

Note that, in the experimentally accessible strain range, GGA gives a qualitatively different prediction from LDA especially for the case of 1-uc. Vertical dashed lines indicate the strain values corresponding to the available substrates. Within the −2–+1.5% range, LDA gives zero (or very small) moment while GGA predicts magnetic phase and the moment is quite large. It should be noted that the zero strain in our calculations is set to be the LDA/GGA-optimized lattice parameters obtained by each XC functional and therefore two values at a given strain value represent the different in-plane lattice parameters; larger in GGA case and smaller in LDA.

As an example, let us first consider the mono-layer SRO113 grown on NdGaO_3_ substrate (dark-blue vertical lines)^32^. Due to the lattice mismatch, this corresponds to the −1.81% of compressive strain situation. For this case, GGA predicts the ferromagnetic spin order while LDA solution is nonmagnetic. The same is true for the case of Sr_2_RuO_4_ substrate^33^ which corresponds to −1.55% compressive strain. Our result therefore raises a question about the predictive power of standard first-principles methodology. On the one hand, it shows that the special care needs to be paid in the first-principles study of correlated oxide thin films and/or heterostructures even with the standard XC energy functionals. On the other, our result calls the further method development which can better describe the electronic correlations and the structural properties. We note that in this kind of systems conventional experimental techniques are often less useful. In this sense, correlated oxide heterostructure and thin film pose a new challenge to the first-principles methodology (see Discussion).

Another notable example is SRO113 film on SrTiO_3_ substrate (−0.38% compressive strain). Contrary to the previous experimental reports^15–18^, there is no clear magnetic-to-nonmagnetic and metal-to-insulator (MI) transition in both of our GGA and LDA results. In 1-uc case, GGA result is ferromagnetic with ~1.4 *μ*_B_/Ru while a significantly smaller magnetic moment of ~0.2 *μ*_B_/Ru is found in LDA (Fig. 2(c)). It may seem inconsistent not only with experiments but also with the previous LDA^16, 20^ calculations since they report the magnetic to non-magnetic transition as the SRO layer thickness is reduced although the critical thickness is still under debate^15–18, 20^. It should be noted however that in the previous LDA^16^ studies, the experimental SrTiO_3_ lattice parameter (3.905 Å) is taken for the calculations. According to our optimized lattice parameters, this corresponds to +0.69% tensile strain for the case of LDA and −2.08% compressive strain for GGA, both of which are significantly different from the experimental situation of −0.38% compressive strain. In fact, our results of +0.69% tensile and −0.38% compressive strain are consistent with the previous calculations^16, 20^. At the correct strain of SRO113 on SrTiO_3_ (−0.38%), the LDA magnetic moment gradually decreases from 3-uc to 1-uc which may be understood as a magnetic to non-magnetic transition observed in experiments. In GGA, however, ferromagnetism survives down to 1-uc thickness. This behavior is double checked by other codes, namely, OpenMX and ecalj.

It is clear that understanding the SRO113 thin film based on 1*a* × 1*a* is quite limited. The tilting and rotation distortions can play important roles in determining the electronic and magnetic property.

### Distorted thin films: $\sqrt{{\bf{2}}}{\boldsymbol{a}}{\boldsymbol{\times }}\sqrt{{\bf{2}}}{\boldsymbol{a}}$ lateral unitcell

The octahedral distortion (tilting and rotation of RuO_6_ cage) and the use of enlarged lateral uc can lead to a qualitatively different ground state solution. The 2-uc-thick SRO113 is found to have FM-M ground state in the strain range of about −3 to +2.5%. At ~3%, G-type-like (*i*.*e*., all the nearest-neighbor couplings are antiferromagnetic) AF-I is stabilized in both LDA and GGA with Ru-site magnetic moment of 0.74 *μ*_B_/Ru (LDA) and 1.51 *μ*_B_/Ru (GGA). It is markedly different from the strained bulk SRO113 for which ferromagnetic ground state is fairly robust over a wide range of strain and G-type AF-I state is not stable^30, 31^.

**Figure 3:**
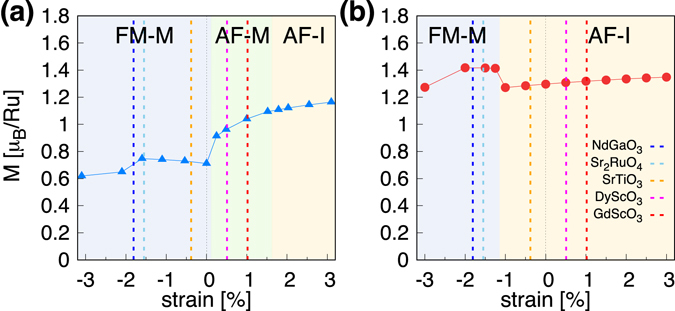
The calculated *M* of 1-uc $\sqrt{2}$ *a* × $\sqrt{2}$ *a* o-SRO113 by (**a**) LDA and (**b**) GGA as a function of strain. The different types of ground configurations (FM-M, AF-M, and AF-I) are presented by background colors. The vertical dashed lines represent the strains realizable by NdGaO_3_ (blue), Sr_2_RuO_4_ (light blue), SrTiO_3_ (orange), DyScO_3_ (magenta), and GdScO_3_ (red) as substrates.

To understand the detailed electronic structure and its relation to the magnetism, we focus on the thinnest case (*i*.*e*., 1-uc thick) in the remaining part of this subsection. The ground state phase diagram as a function of strain is presented in Fig. 3; (a) LDA and (b) GGA. The first thing to be noted is that both XC functionals predict FM-M and AF-I ground state in the large compressive and large tensile strain region, respectively. The size of magnetic moment is generally larger in GGA than LDA, which is the same feature found in the previous cases of bulk and undistorted thin film.

In the experimentally more accessible range, however, LDA and GGA give qualitatively different solutions. In the GGA phase diagram (Fig. 3(b)), AF-I is the ground state around zero strain region (≤±1% strain) while LDA predicts either FM-M or antiferromagnetic metal (AF-M). The AF-M phase of LDA (which is absent in GGA phase diagram) is attributed to the smaller energy splitting between *d*_*xy*_ and *d*_*yz*,*zx*_ states near E_F_. Thus, for the case of SrTiO_3_^32^, DyScO_3_^32^ or GdScO_3_^14^ substrate, we have two different predictions regarding the ground state property from two standard XC functionals. It therefore raises a serious question about the predictive power of the current first-principles method to describe the correlated oxide thin film and/or heterostructure for which the experimental information is often limited. Further investigation and development are urgently necessary.

**Figure 4:**
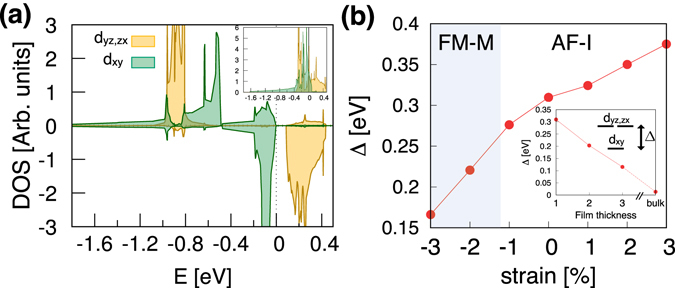
(**a**) The Ru-t_2*g*_ DOS by GGA for 1-uc o-SRO113 at −0.5% strain. The positive and negative DOS represent the up- and down-spin parts, respectively. The Fermi level is set to zero. The non-spin-polarized DOS is shown in the inset. (**b**) The calculated crystal-field splitting, Δ, for 1-uc o-SRO113 by GGA as a function of strain. The inset shows Δ as a function of film thickness (the number of layers) at 0% strain.

Noticeable is our GGA result of AF-I ground state for SrTiO_3_ substrate (see Fig. 3(b)). We emphasize that this AF-I phase has never been achieved before by LDA and GGA^16, 20–22^. Since AF-I has previously been obtained only by DFT + *U* ^21, 22^ or DFT + DMFT (DFT + dynamical mean-field theory)^24^ and it can be consistent with the recent experiments reporting an insulating state carrying no net moment^17, 18^, it has been discussed that the insulating gap of 1-uc SRO113 is opened by on-site Coulomb correlation^24^. However, Fig. 4(a) clearly shows the band-gap without the explicit inclusion of *U* while the gap size is smaller than that of DMFT (${{\rm{E}}}_{{\rm{g}}}^{{\rm{DMFT}}}$ = 1.0 eV)^24^. Our result therefore implies that the insulating ground state of mono-layer SRO113 is not just attributed to the local Coulomb physics within Ru-*d* electrons, but the homogeneous electron approximation can describe the gap opening. It also shows that the careful consideration of lattice effect is important to simulate the experimental situation of complex oxides.

A common trend found in both LDA and GGA phase diagram is that the tensile strain makes system antiferromagnetic while the compressive strain favors ferromagnetic. It can be simply understood by defining an energy level difference between Ru-*d*_*yz*,*zx*_ and *d*_*xy*_ state: ${{\rm{\Delta }}}^{{\rm{LDA}}/{\rm{GGA}}}\equiv {\varepsilon }_{yz,zx}^{{\rm{LDA}}/{\rm{GGA}}}-{\varepsilon }_{xy}^{{\rm{LDA}}/{\rm{GGA}}}$. It is straightforward to compute *ε* by using the standard technique of maximally localized Wannier function^34, 35^. At 0% strain, Δ^LDA^ = 0.33 and Δ^GGA^ = 0.31 eV. As the in-plane lattice parameter gets smaller (more compressive strain), Δ decreases to 0.15 (LDA) and 0.17 (GGA) eV at ~−3% while under tensile strain it becomes larger to 0.40 (LDA) and 0.38 (GGA) eV at ~+3%. The same trend is also observed in the 2-uc-thick film; Δ = 0.21 eV (LDA) and 0.20 eV (GGA) at 0% strain while Δ = 0.27 eV (LDA) and 0.27 eV (GGA) at ~+3% strain where AF-I is stabilized. The calculated results of Δ^GGA^ is presented in Fig. 4(b). In the large Δ limit (large tensile strain regime), the separation between *ε*_*yz*,*zx*_ and *ε*_*xy*_ is large and it approximately corresponds to the (two-band) half-filled case within the ionic picture of Ru^4+^ (see inset of Fig. 4(a)) as in the case of Ca_2_RuO_4_^36^. Thus the antiferromagnetic spin order is naturally stabilized via superexchange. On the other hand, the small Δ limit (compressive strain) basically corresponds to the bulk SRO113 regime in which ferromagnetic order is stable.

The inset of Fig. 4(b) shows the calculated Δ^GGA^ as a function of SRO thickness. A decreasing trend is clearly noticed as the number of layers increases. Since this is a qualitatively consistent trend with the magnetic transition observed in the several experiments as a function of layer thickness, our result implies that the Δ plays an important role in the thickness-dependent transition. Therefore it is probably not understood solely from N(E_F_) and Stoner’s criterion^16^, but rather comparable with the case of SrVO_3_/SrTiO_3_ in which the lifted orbital degeneracy plays an important role to induce the MI transition^37, 38^. We do not find any systematic trend in N(E_F_) over the range of strain. In the sense that the FM-M to AF-I transition occurs in between 2- and 1-uc thickness, our GGA result is not in quite good agreement with experiments which report the transitions in between 5- and 4-uc^15^, or 4- and 3-uc^17, 18^, or 3- to 2-uc^16^. Further inclusion of electron correlations might be needed to correctly describe the Δ and other electronic properties^19, 38–43^.

It might be informative to see DFT + *U* ^44^ results. In spite of its static Hartree-Fock nature, it has been used to study SRO113^21, 22^. With a fixed *U* value to a recent cRPA (constrained random phase approximation) result (*U* = 3.5 eV^24^) and at a −0.5% compressive strain (close to SrTiO_3_ value), AF-I phase is found to be the ground state. For 1-uc thickness, E_g_ = 1.1 (1.6) eV and *M* = 1.47 (1.60) *μ*_B_/Ru in LDA + *U* (GGA + *U*). In 2-uc thickness, E_g_ = 0.9 (1.3) eV and *M* = 1.41 (1.57) *μ*_B_/Ru in LDA + *U* (GGA + *U*).

## Discussion

Our results show that the prediction of the magnetic ground state of complex oxide thin film or heterostructure is a challenge to the current first-principles methodology. Without further information such as structural property and/or magnetic order (usually known from experiment in the case of bulk), it is difficult to make a prediction for thin film SRO113. Note that LDA and GGA are not a bad choice to describe the bulk SRO113 in the sense that both predict the correct FM-M ground state. Although some detailed features in the electronic structure are not well captured by LDA or GGA (*e*.*g*., Hubbard-like state ~−1.3 eV below the Fermi energy; see, ref. 45), these standard approximations provide reasonable descriptions of this metallic bulk material. Arguably, they are better than other improved but static methods to take orbital-dependent correlations into serious account such as DFT + *U* ^11^. It is therefore surprising to see such a large difference between LDA and GGA in this system.

We first note that this difficulty is related to the complex nature of correlated oxides in which the spin, orbital, and lattice degree of freedom are tightly coupled to each other. On the one hand, it is the reason why the parameter-free first-principles techniques are strongly requested. On the other hand, this complexity makes the first-principles description be a greater challenge. Note that an improved description of electronic correlations cannot immediately be the right answer unless it can simultaneously give the better description of the other degrees of freedom such as lattice. For example, if we resort to DMFT approximation to get a better electronic structure of ruthenates, we have to describe the force and the lattice relaxation simultaneously at the same level. This development is technically non-trivial^46–48^. Similar issues and related intrinsic ambiguities can also be found in other techniques such as SIC-DFT (self-interaction corrected DFT) and hybrid functional.

Here it is instructive to recall that the metallic magnetism has been a challenge to the first-principles calculation^49–52^. A classical example is bcc Fe for which minimum energy solution of LDA is paramagnetic hcp phase (with the optimized lattice parameter)^49^ unlike Co and Ni^53^. In other words, without *a priori* knowledge of ferromagnetism, the situation of bcc Fe is similar with SRO113 thin film; paramagnetic ground state by LDA and ferromagnetic in GGA. Since the use of experimental lattice parameter gives the correct ferromagnetic solution in both LDA and GGA, bcc Fe is an easier case in the practical sense. In SRO113 thin film, on the other hand, the limitation of computation method is magnified due to no *a priori* information of structure and magnetic order.

**Figure 5:**
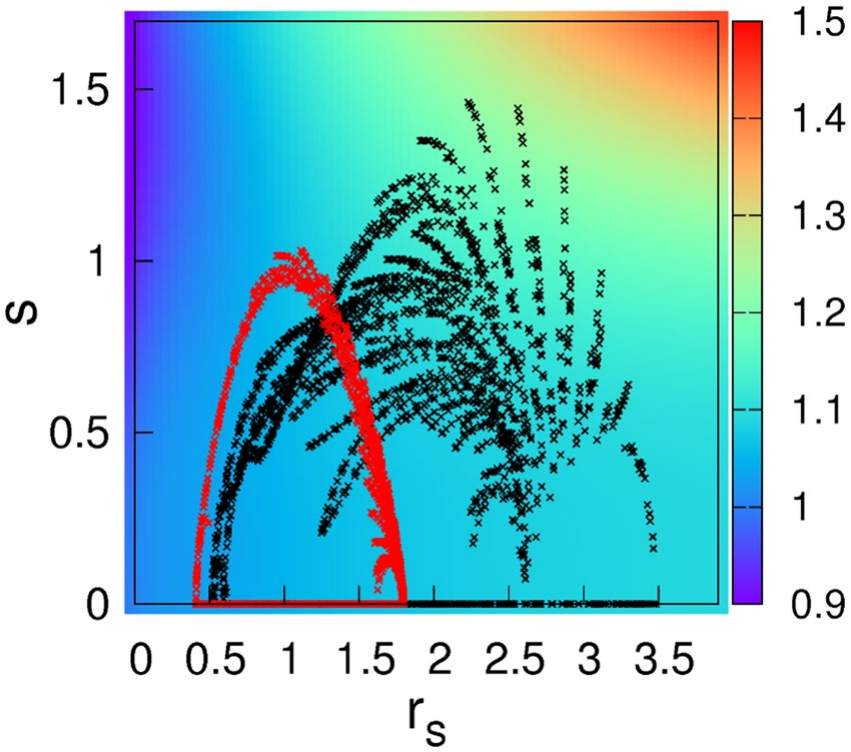
The calculated *α*_XC_ for c-SRO113 (black crosses) and bcc Fe (red crosses). About 85000 real-space grid points are obtained from the self-consistently converged electronic structure within LDA. Experimental lattice parameters are used. *ζ* is fixed to 0.02 which is the average value of c-SRO113. The *α*_XC_ change is negligible in the range of 0 < *ζ* < 0.5.

A more detailed nature of enhanced ferromagnetism by GGA functional can be seen in Fig. 5 where the PBE (Perdew-Burke-Zunger) correction of spin-polarized energy density with respect to LDA is presented as a function of inverse density (*r*_*s*_) and density gradient (*s*) for the relative spin-polarization (*ζ*). The calculated values of

3 $${\alpha }_{{\rm{XC}}}\equiv \frac{{\rm{\Delta }}{\varepsilon }_{{\rm{XC}}}^{{\rm{PBE}}}({r}_{s},\zeta ,s)}{{\rm{\Delta }}{\varepsilon }_{{\rm{XC}}}^{{\rm{LDA}}}({r}_{s},\zeta )}$$

are represented by color. Here ${\rm{\Delta }}{\varepsilon }_{{\rm{XC}}}^{{\rm{PBE}}}({r}_{s},\zeta ,s)={\varepsilon }_{{\rm{X}}}^{{\rm{unif}}}({r}_{s},\mathrm{0)[}{F}_{{\rm{XC}}}({r}_{s},\zeta ,s)-{F}_{{\rm{XC}}}({r}_{s},0,s)]$ and ${\rm{\Delta }}{\varepsilon }_{{\rm{XC}}}^{{\rm{LDA}}}({r}_{s},\zeta )=$ $f(\zeta )[{\varepsilon }_{{\rm{XC}}}^{{\rm{unif}}}({r}_{s},\mathrm{1)}-{\varepsilon }_{{\rm{XC}}}^{{\rm{unif}}}({r}_{s},\mathrm{0)]}$ refer to the spin-polarized part of XC energy density within PBE and LDA, respectively. For the definitions of *ε*, *r*_*s*_, *ζ*, *s*, *F*_XC_(*r*_*s*_, *ζ*, *s*), and *f*(*ζ*), we follow the original PBE^54^ and LDA-PZ (Perdew-Zunger)^55^ papers. By definition, *α*_XC_ represents the energy gain by GGA-PBE over LDA. In the yellow-red colored region of Fig. 5, GGA-PBE prefers the magnetic solution more than LDA while in the dark blue region LDA-like solution is favored. About 85000 real-space grid points (using OpenMX code) are plotted in this map for bcc Fe (red-colored points) and c-SRO113 (black-colored points), respectively. It is clear that both bcc Fe and c-SRO113 have large portion of grid points in the region of *α*_XC_ larger than unity. It is responsible for the larger spin splitting (~$\partial {\rm{\Delta }}{\varepsilon }_{{\rm{XC}}}/\partial \zeta$) and thus the moment formation enhanced by PBE correction.

Since many interesting aspects of complex oxide films and interfaces are directly or indirectly related to the metallic magnetism^56–60^, it is strongly requested to develop more reliable computation methods. One promising aspect is the same overall trend given by LDA and GGA. For example, the moment is suppressed by compressive strain in the case of 1*a* × 1*a* lateral uc (Fig. 2(a,b)). FM-M and AF-I phase in the large compressive and large tensile strain, respectively, are also consistently reproduced by LDA and GGA (Fig. 3). Further investigation of parameterization hopefully gives a way to improve approximations.

## Summary

From a systematic first-principles investigation, we elucidate the controversies in the previous calculations and experiments. A part of issues has been clarified including the thickness- and strain-dependent phase transition as well as the ground state property of monolayer SRO113. The role of strain, correlation and lattice distortions are explored in detail. Applicability of LDA and GGA has been discussed in the realistic material context by using Landau free energy analysis and magnetization-energy-ratio plot. While the overall feature is commonly reproduced by both approximations, they give qualitatively different predictions for the case of thin film in the experimentally relevant region of strain. Our work provides useful information not only for ruthenates and other correlated oxides, but also for further first-principles studies in the related systems such as metallic magnetism.

## Methods

We choose LDA parameterized by Perdew and Zunger^55^ and GGA by Perdew, Burke, and Ernzerhof^54^ as two representative XC functionals. The data obtained by these two functionals is presented as our main results and discussed in a comparative way. We used projector-augmented-wave (PAW) method^61^ as implemented in the VASP software package^62^. While VASP-PAW is used to obtain our main results, we also double checked some cases with other methods, confirming that our conclusions are valid. The other codes we used include OpenMX^63^, which is based on the norm-conserving pseudopotential and pseudoatomic orbitals, and ecalj^64^ based on the full-potential ‘LMTO’ or ‘PMT’ basis^65^.

Tetrahedron method with Blöchl correction has been used for Brillouin zone integration^66^, which we found is important to achieve the enough accuracy. In some cases the use of Gaussian broadening is found to give qualitatively different predictions depending on the broadening value. For bulk structure optimizations, 12 × 12 × 12 and 8 × 8 × 6 **k**-points were used for 5-atom uc of c-SRO113 and 20-atom uc of o-SRO113, respectively. The force criterion of 1 meV/Å was adopted for structural optimization. This choice of **k** meshes and the 500 eV energy cutoff were found to be satisfactory from our convergence test. For the electronic structure calculations, however, we took a denser **k**-grid of 16 × 16 × 16 for c-SRO113 and 12 × 12 × 10 for o-SRO113 to calculate DOS for the given optimized structures. To simulate SRO113 thin films, we considered the 3-uc, 2-uc, and 1-uc slab geometries terminated with SrO layers which is known to be the case in experimental situations^13^. The **k**-grids used for slab calculations were 12 × 12 × 2 for c-SRO113 and 8 × 8 × 2 for o-SRO113. The vacuum thickness is ≥15 Å which is large enough to simulate the experimental situation. The Ru radius is set to 1.402 Å and the Ru moment dependence on this setting is negligible (~0.01 *μ*_B_).

Epitaxial strain is one of the key control parameters in the thin film growth. It is therefore of crucial importance to determine how to simulate the in-plane lattice parameter corresponding to the experimental situation. Note that this is a non-trivial issue because LDA- and GGA-optimized lattice parameters are both in general different from the experimental values. While the former tends to underestimate the lattice parameter, the latter overestimates (see Table 1). Thus, the naive choice of a XC functional or taking experimental lattice value can easily be misleading. Previous studies are not quite consistent in this regard. In the current study, we take the optimized lattice constant with a given XC functional as the reference value (*i*.*e*., zero strain) and define the compressive/tensile strain value with respect to it. This can be the most reasonable way to simulate the experiments and can serve as a solid reference for the future study. This point should be kept in mind when our results are compared with the previous calculation results^16, 22^.

## References

[1] Ohtomo A, Muller DA, Grazul JL, Hwang HY (2002). Artificial charge-modulationin atomic-scale perovskite titanate superlattices. Nature.

[2] Dawber M, Rabe KM, Scott JF (2005). Physics of thin-film ferroelectric oxides. Rev. Mod. Phys..

[3] Mannhart J, Blank DHA, Hwang HY, Millis AJ, Triscone J-M (2008). Two-dimensional electron gases at oxide interfaces. MRS bulletin.

[4] Chakhalian J, Freeland JW, Millis AJ, Panagopoulos C, Rondinelli JM (2014). *Colloquium*: Emergent properties in plane view: Strong correlations at oxide interfaces. Rev. Mod. Phys..

[5] Stemmer S, Allen SJ (2014). Two-dimensional electron gases at complex oxide interfaces. Annu. Rev. Mater. Res..

[6] Fennie CJ, Rabe KM (2006). Magnetic and electric phase control in epitaxial EuTiO_3_ from first principles. Phys. Rev. Lett..

[7] Lee JH (2010). A strong ferroelectric ferromagnet created by means of spin-lattice coupling. Nature.

[8] Chaloupka J, Khaliullin G (2008). Orbital order and possible superconductivity in LaNiO_3_/LaMO_3_ superlattices. Phys. Rev. Lett..

[9] Hansmann P (2009). Turning a nickelate fermi surface into a cupratelike one through heterostructuring. Phys. Rev. Lett..

[10] Han MJ, Wang X, Marianetti CA, Millis AJ (2011). Dynamical mean-field theory of nickelate superlattices. Phys. Rev. Lett..

[11] Anisimov VI, Aryasetiawan F, Lichtenstein AI (1997). First-principles calculations of the electronic structure and spectra of strongly correlated systems: the LDA + U method. J. Phys.: Condens. Matter.

[12] Kotliar G (2006). Electronic structure calculations with dynamical mean-field theory. Rev. Mod. Phys..

[13] Koster G (2012). Structure, physical properties, and applications of SrRuO_3_ thin films. Rev. Mod. Phys..

[14] Thompson J (2016). Enhanced metallic properties of SrRuO_3_ thin films via kinetically controlled pulsed laser epitaxy. Appl. Phys. Lett..

[15] Toyota D (2005). Thickness-dependent electronic structure of ultrathin SrRuO_3_ films studied by *in situ* photoemission spectroscopy. Appl. Phys. Lett..

[16] Chang YJ (2009). Fundamental thickness limit of itinerant ferromagnetic SrRuO_3_ thin films. Phys. Rev. Lett..

[17] Xia J, Siemons W, Koster G, Beasley MR, Kapitulnik A (2009). Critical thickness for itinerant ferromagnetism in ultrathin films of SrRuO_3_. Phys. Rev. B.

[18] Ishigami K (2015). Thickness-dependent magnetic properties and strain-induced orbital magnetic moment in SrRuO_3_ thin films. Phys. Rev. B.

[19] Georges A, de Medici L, Mravlje J (2013). Strong correlations from Hund’s coupling. Annu. Rev. Condens. Matter Phys..

[20] Rondinelli JM, Caffrey NM, Sanvito S, Spaldin NA (2008). Electronic properties of bulk and thin film SrRuO_3_: Search for the metal-insulator transition. Phys. Rev. B.

[21] Mahadevan P, Aryasetiawan F, Janotti A, Sasaki T (2009). Evolution of the electronic structure of a ferromagnetic metal: Case of SrRuO_3_. Phys. Rev. B.

[22] Gupta K, Mandal B, Mahadevan P (2014). Strain-induced metal-insulator transition in ultrathin films of SrRuO_3_. Phys. Rev. B.

[23] Verissimo-Alves M, García-Fernández P, Bilc DI, Ghosez P, Junquera J (2012). Highly confined spin-polarized two-dimensional electron gas in SrTiO_3_/SrRuO_3_ superlattices. Phys. Rev. Lett..

[24] Si L, Zhong Z, Tomczak JM, Held K (2015). Route to room-temperature ferromagnetic ultrathin SrRuO_3_ films. Phys. Rev. B.

[25] Mazin II, Singh DJ (1997). Electronic structure and magnetism in Ru-based perovskites. Phys. Rev. B.

[26] Han Q, Dang HT, Millis AJ (2016). Ferromagnetism and correlation strength in cubic barium ruthenate in comparison to strontium and calcium ruthenate: A dynamical mean-field study. Phys. Rev. B.

[27] Jeong DW (2013). Temperature evolution of itinerant ferromagnetism in SrRuO_3_ probed by optical spectroscopy. Phys. Rev. Lett..

[28] Mackenzie AP, Maeno Y (2003). The superconductivity of Sr_2_RuO_4_ and the physics of spin-triplet pairing. Rev. Mod. Phys..

[29] Carlo JP (2012). New magnetic phase diagram of (Sr, Ca)_2_RuO_4_. Nat. Mater..

[30] Zayak AT, Huang X, Neaton JB, Rabe KM (2006). Structural, electronic, and magnetic properties of SrRuO_3_ under epitaxial strain. Phys. Rev. B.

[31] Zayak AT, Huang X, Neaton JB, Rabe KM (2008). Manipulating magnetic properties of SrRuO_3_ and CaRuO_3_ with epitaxial and uniaxial strains. Phys. Rev. B.

[32] Dirsyte R (2011). Impact of epitaxial strain on the ferromagnetic transition temperature of SrRuO_3_ thin films. Thin Solid Films.

[33] Anwar MS (2015). Ferromagnetic SrRuO_3_ thin-film deposition on a spin-triplet superconductor Sr_2_RuO_4_ with a highly conducting interface. Appl. Phys. Express.

[34] Marzari N, Mostofi AA, Yates JR, Souza I, Vanderbilt D (2012). Maximally localized Wannier functions: Theory and applications. Rev. Mod. Phys..

[35] Mostofi AA (2014). An updated version of wannier90: A tool for obtaining maximally-localised Wannier functions. Comput. Phys. Commun..

[36] Liebsch A, Ishida H (2007). Subband filling and mott transition in Ca_2−*x*_Sr_*x*_RuO_4_. Phys. Rev. Lett..

[37] Yoshimatsu K (2010). Dimensional-crossover-driven metal-insulator transition in SrVO_3_ ultrathin films. Phys. Rev. Lett..

[38] Zhong Z (2015). Electronics with correlated oxides: SrVO_3_/SrTiO_3_ as a mott transistor. Phys. Rev. lett..

[39] Poteryaev AI (2007). Enhanced crystal-field splitting and orbital-selective coherence induced by strong correlations in V_2_O_3_. Phys. Rev. B.

[40] Tomczak JM, van Schilfgaarde M, Kotliar G (2012). Many-body effects in iron pnictides and chalcogenides: Nonlocal versus dynamic origin of effective masses. Phys. Rev. Lett..

[41] Han MJ, Kino H, Kotani T (2014). Quasiparticle self-consistent GW study of LaNiO_3_ and LaNiO_3_/LaAlO_3_ superlattice. Phys. Rev. B.

[42] Jang SW, Kotani T, Kino H, Kuroki K, Han MJ (2015). Quasiparticle self-consistent GW study of cuprates: electronic structure, model parameters, and the two-band theory for T_c_. Sci. Rep..

[43] Ryee S, Jang SW, Kino H, Kotani T, Han MJ (2016). Quasiparticle self-consistent GW calculation of Sr_2_RuO_4_ and SrRuO_3_. Phys. Rev. B.

[44] Dudarev SL, Botton GA, Savrasov SY, Humphreys CJ, Sutton AP (1998). Electron-energy-loss spectra and the structural stability of nickel oxide: An LSDA + U study. Phys. Rev. B.

[45] Takizawa M (2005). Manifestation of correlation effects in the photoemission spectra of Ca_1−*x*_Sr_*x*_RuO_3_. Phys. Rev. B.

[46] Leonov I, Anisimov VI, Vollhardt D (2014). First-principles calculation of atomic forces and structural distortions in strongly correlated materials. Phys. Rev. Lett..

[47] Park H, Millis AJ, Marianetti CA (2014). Computing total energies in complex materials using charge self-consistent DFT + DMFT. Phys. Rev. B.

[48] Haule K, Birol T (2015). Free energy from stationary implementation of the DFT + DMFT functional. Phys. Rev. Lett..

[49] Asada T, Terakura K (1992). Cohesive properties of iron obtained by use of the generalized gradient approximation. Phys. Rev. B.

[50] Aguayo A, Mazin II, Singh DJ (2004). Why Ni_3_Al is an itinerant ferromagnet but Ni_3_Ga is not. Phys. Rev. Lett..

[51] Mazin II, Johannes MD, Boeri L, Koepernik K, Singh DJ (2008). Problems with reconciling density functional theory calculations with experiment in ferropnictides. Phys. Rev. B.

[52] Ortenzi L, Mazin II, Blaha P, Boeri L (2012). Accounting for spin fluctuations beyond local spin density approximation in the density functional theory. Phys. Rev. B.

[53] Moroni EG, Kresse G, Hafner J, Furthmüller J (1997). Ultrasoft pseudopotentials applied to magnetic Fe, Co, and Ni: From atoms to solids. Phys. Rev. B.

[54] Perdew JP, Burke K, Ernzerhof M (1996). Generalized gradient approximation made simple. Phys. Rev. Lett..

[55] Perdew JP, Zunger A (1981). Self-interaction correction to density-functional approximations for many-electron systems. Phys. Rev. B.

[56] Takahashi KS, Kawasaki M, Tokura Y (2001). Interface ferromagnetism in oxide superlattices of CaMnO_3_/CaRuO_3_. Appl. Phys. Lett..

[57] Grutter AJ (2013). Interfacial ferromagnetism in LaNiO_3_/CaMnO_3_ superlattices. Phys. Rev. Lett..

[58] Li L, Richter C, Mannhart J, Ashoori RC (2011). Coexistence of magnetic order and two-dimensional superconductivity at LaAlO_3_/SrTiO_3_ interfaces. Nat. Phys..

[59] Bert JA (2011). Direct imaging of the coexistence of ferromagnetism and superconductivity at the LaAlO_3_/SrTiO_3_ interface. Nat. Phys..

[60] Hoffman J (2013). Charge transfer and interfacial magnetism in (LaNiO_3_)_*n*_/(LaMnO_3_)_2_ superlattices. Phys. Rev. B.

[61] Blöchl PE (1994). Projector augmented-wave method. Phys. Rev. B.

[62] Kresse G, Joubert D (1999). From ultrasoft pseudopotentials to the projector augmented-wave method. Phys. Rev. B.

[63] Ozaki T (2003). Variationally optimized atomic orbitals for large-scale electronic structures. Phys. Rev. B.

[64] The electronic structure calculation package, ‘ecalj’. https://github.com/tkotani/ecalj.

[65] Kotani T, Kino H, Akai H (2015). Formulation of the augmented plane-wave and muffin-tin orbital method. J. Phys. Soc. Jpn..

[66] Blöchl PE, Jepsen O, Andersen OK (1994). Improved tetrahedron method for brillouin-zone integrations. Phys. Rev. B.

[67] Jones CW, Battle PD, Lightfoot P, Harrison WTA (1989). The structure of SrRuO_3_ by time-of-flight neutron powder diffraction. Acta Crystallogr. Sec. C: Cryst. Struct. Commun..

